# Atrophic Rhinitis

**Published:** 1892-07

**Authors:** Charles D. Roy

**Affiliations:** Assistant to Chair of Diseases of the Eye, Ear, Nose, etc., in Southern Medical College, Atlanta, Georgia


### ATROPHIC RHINITIS.

BY

CHARLES D. ROY, A. B., M. D.,

Assistant to Chair of Diseases of the Eye, Ear, Nose, etc., in Southern Medical College,

ATLANTA, GEORGIA.

The word catarrh strikes as much terror in the minds of the American people to-day as once did the "Black Hole" of Calcutta in those of the English. This is largely due to the bombastic parade of quack advertisements in our daily papers which inflict upon an unsuspecting and eagerly-seeking public a dissertation upon this, as they would have us believe, the most loatheful disease to which man is heir. Such advertisements are nothing if not sweeping in their statements, for there is never omitted a symptom which is not possessed by some poor benighted individual, or who has at least not only had one but a dozen of those enumerated. Even while I am writing this there appears in the columns of one of our large dailies the following: "A

ringing noise in the ears, headache, deafness, eyes weak; obstruction of the nose, discharge falling into the throat, sometimes profuse, watery and acrid; at others thick, tenacious, bloody and putrid; offensive breath; smell and taste impaired and general debility. That's catarrh." What a benighted race we are, for according to the above there is not one of us who is not afflicted with the dreaded disease! Unfortunately it is the uneducated public who receive the benefits of these fallacious statements, and the latter can only be eradicated by the thorough teachings of honest physicians. Catarrh is a word that I wish could be banished from medical nosology, for while physicians understand its meaning and the conditions to which it applies, the laity associate the term only with the worst diseases of the nasal organs. The word, as we know, comes from two Greek terms, *kata* and *rheo*, meaning a flowing from, and its full definition as given in *Foster's Medical Dictionary* is: "A simple inflammation of any mucous membrane in which the blood vessels become engorged, and swelling takes place from the exudation of serum." Dunglison, Quain, Thomas, and others, all practically agree with the above definition. From this we see how unmedical it is to limit the meaning of this term only to diseased conditions of the nasal cavities, for since mucous membranes exist in other portions of the body each is liable to be attacked by the same pathological conditions. We speak of catarrh of the eyes, of the bladder, of the urethra, of the vagina, of the bowels, each conveying to the physician a definite morbid state, but were we to speak of the same to the laity they would probably be surprised to hear that such a condition ever existed. Again, the word catarrh in nasal diseases is inapt, for by its definition a catarrhal state of the nasal mucous membrane is fortunately of rare occurrence. The most pronounced type of this condition which we find in this locality is an acute rhinitis or, as is generally known, a cold in the head, but how few patients ever come to the physician in this state and say that they are suffering with catarrh. The word, as we have seen, signifies a flow, an increased amount of secretion, but in nasal stenosis due to a hypertrophic condition of the mucous membrane, the most common of all nasal troubles, we find a seemingly diminished amount of secretion. Hence it is that the word has been so perverted from its original meaning that it carries with it in the minds of the public a feeling of disgust and awe, and for these reasons it should be expunged from our medical vocabulary when addressing patients. The bombastic quacks and patent medicine fiends who infest pre-eminently the Gate City of the South, tell the public that catarrh is a blood disease, and as such, they must take medicine internally if they wish to be cured, thus exacting from innocent people their hard earned dollars with which to pay for fluid not one-hundreth part as purifying as yon mountain stream. While we might theorize as to the probable influence which the trophic and vaso-motor centers exert over the nasal mucous membrane, I am sure that there is no rhinologist who does not consider local changes and local medication as paramount in the successful treatment of this disease.

The term atrophic rhinitis, which I use as expressive of the pathological condition found, is the one most generally used, but like many other terms in medicine, it is open to criticisms. Sajous uses as synonyms the expressions dry catarrh, atrophic catarrh. Lennox Browne, of London, uses the term atrophic rhinitis, but distinguishes it into the simple or non-fetid, specific or fetid. Dr. Meran, of Italy, in the *Arch. de Laryngol. de Rhinol*, etc., uses the term offensive hypersecretory rhinitis when there is simply the offensive discharge without the atrophy.

Ozæna is a term often erroneously used in medical text-books for representing this form of disease, but we must remember that the word is significant of a symptom only, and one which does not always exist. Ozæna, as its name implies, means a fetid odor, usually with an accompanying discharge from the nasal cavities and is most constant when the disease is due to syphilis or tuberculosis. In my clinical work I am accustomed to divide simple atrophic rhinitis into two forms: (1) Mixed atrophy; (2) complete atrophy. The distinction is not absolute, for it depends upon the period at which the mixed form is seen, since the latter eventually emerges into the complete atrophic state. The word atrophic, as used, designates that condition of the mucous membrane and glands lining the nasal cavities found in this diseased condition. If such a nasal cavity be examined, the mucous membrane covering its walls, and especially that over the turbinates will be found shrunken and of almost horny hardness, being destitute of that generally moistened condition found when lubricated by the normal mucous secretion.

The mucous glands being destroyed, what secretion as does exist rapidly desiccates and great flakes of these dried masses are seen covering its surface, adhering with great tenacity to the surrounding walls. The above description represents the true atrophic variety, but there is a form to which the term is sometimes incorrectly applied, where the membrane to all appearances is normal, but there is an undue amount of purulent, fetid secretion, probably from the accessory cavities, especially in that form which Woakes, of London, calls necrosing ethmoiditis. In the mixed form the pathological anatomy differs in extent only, the atrophic portion most commonly being confined to the posterior portions of the turbinates and surrounding mucous membrane, while the anterior portion of the nasal cavities is usually in a hypertrophic state. The treatment of this mixed form I have found very tedious and it is well to early recognize its existence. The etiology of atrophic rhinitis or chronic atrophy of the glands of mucous membrane of the nasal cavities is still obscure, and in the minds of some of the best observers still undecided. This is a point on which most distinguished rhinologists differ. Dr. Sajous, of Philadelphia, with the late Sir Morrell Mackenzie, probably expresses a large consensus of opinion when he says that atrophy appears to be always a secondary affection. Lennox Browne, of London, says that atrophy is often primary, due, he thinks, to a morbid diathetic state of the system. I myself am a strong believer in the atrophic form following the hypertrophic, since the numerous cases of mixed atrophy are strong points in its favor, and because the hypertrophied state is well calculated to be a direct causal factor. Dr. Bosworth, of New York, does not think that atrophy is ever caused from a previous hypertrophy. Of course there could be no disease without there being advocates of a germ theory, and this one is no exception to the rule. Its strongest advocate is Lowenberg, but as yet the germ idea has not met with strong support. Dr. Jonathan Wright, of Brooklyn, one of the best practical writers upon this subject, believes that what organisms have been found in the secretions can only account for the odor which is usually present. Dr. Jno. McKenzie, of Baltimore, holds that there is a close relationship between atrophic rhinitis and the sexual organs, and I myself have noticed that the nasal trouble is worse at the menstrual periods. Lennox Browne, above quoted, calls attention to two very important predisposing factors: (1) Abnormal patency of the anterior nares; (2) the constant inhalation of an unsanitary atmosphere, including, of course, irritating particles, as coal dust, a fact well worth the attention of the city sanitary officers. This form of nasal trouble is more usually confined to those about the age of puberty, especially among young girls, so much so that Voltalini calls it a woman's disease. I have also seen it frequently in the very young, and less commonly after adult life, save the normal shrinking, which usually takes place in senile life. While unsanitary surroundings are strong predisposing factors, the children of Crœsus seem to suffer as often as do the dwellers in the humble huts. Were the success in the treatment in this nasal disease as easy of accomplishment as is its diagnosis, the physician, as well as the patient, would be blessed indeed, but unfortunately, such is not the case. The symptoms of this condition are familiar, no doubt to many of you who do, and to those of you who do not, use the nasal speculum, especially in such cases where the odor is no poet's fancy. The subjective and objective symptoms are most prominent. The patient is aware of an undue patency of the nasal fossæ, for with the respiratory act the air passes through these cavities as if through unobstructed cylinders. He is troubled with the tough, scabby secretions, which are continually blown from the nose. A feeling of freedom from these secretions is seldom his lot, even though the anterior and posterior exits seem never derelict in their duty. As a usual rule there is an accompanying odor, both subjective and objective, subjective provided the olfactory region has not been destroyed in the pathological process. Coupled with these symptoms and paramount to all is its moral effect upon the patient in causing him to feel that he is not only a discomfort to himself, but even to those around him. The objective symptoms are no less marked. If you look into such a nose, the first thing which strikes the observer's eye is the unnatural roominess of the cavities. The mucous membrane will be found atrophied and shrunken, and with it sometimes even the turbinate bones. Clinging to the walls will be these large flakes of dried secretion, which will sometimes require even the aid of forceps to disengage them from their attachment, after leaving a surface bleeding and in a circumscribed necrosed condition. If it be the mixed form, one will usually find the anterior portions of the inferior turbinates in a hypertrophic state, and on closer observations the posterior regions of the same and the contiguous mucous membrane will be found atrophied and coated with this tenacious secretion. In this latter variety the secretion will be prone to gather in the post nasal space, and instead of having its exit anteriorly, the patient will be troubled with a continual dropping in the throat. The secretion here referred to is not such as is found in the catarrhal condition of the antrum, frontal sinuses, or adjacent cavities, but such as is found in true atrophy. Another prominent and distressing symptom, which is usually present in the majority of cases of true atrophic rhinitis, is the odor. This is a distinct entity and has no analogue in nature to which it could be likened. It is rather a sickening odor, and once experienced can never be forgotten. It is so permeating that often I have detected it as soon as the patient had entered the office door. As a usual rule the olfactory region has been so destroyed that the patients themselves are not cognizant of this symptom. Accompanying this morbid state of the nasal cavities is the dry, glazed atrophic state of the pharyngeal mucous membrane, and even that of the larynx suffers in the strife.

While the above remarks have in no way exhausted the pathological manifestations of this disease, the most important feature for us to consider to-night is the best method of treatment. One must fully understand the pathology of the disease before he can treat it rationally and successfully, and above all, before he is able to tell the patient what he may expect. Honesty, even in medical parlance, is too often forgotten. The physician who tells his patient with an easy *suaviter in modo* that he can cure him, not only does the patient an injustice, but himself a greater one. Nor should he ever limit himself as to time when the patient asks him to state the period necessary for treatment. I always tell such what they are to expect and never desire to treat a person who wants a positive answer as to the length of time they must be under treatment. I say to all such that they can never hope to be cured in so far as the cure means a regeneration of the walls of the nasal cavities to their original or normal state. That they can be benefited I never hesitate to affirm; that is, that they can be placed in a condition where they will be a comfort to themselves and no discomfort to others, but that the treatment must be for months and perhaps years. One word is the groundwork for its successful treatment, *cleanliness*, and until the case has been gotten thoroughly under control by the physician, he must systematically conduct the treatment himself in the office, with the auxiliary aid of the patient at home. First of all the nasal cavities must be thoroughly freed from all secretion, and this can scarcely ever be done satisfactorily at first, with simply a spray. If the mucous membrane is dry and the crusts hardened, the parts must first be thoroughly lubricated by means of a spray of aristol in albolene (10 grains to the ounce). After this is done and the patient has evacuated the contents as far as his expiratory powers are able, there will still remain a considerable amount of secretion too tenacious to be thus expelled, and it is then that the soft cotton probe can so successfully be used in dislodging the offending masses.

Recently I have used a spray of peroxide of hydrogen 1 part to 3 parts of water, on account of its penetrating properties, and thus far I may say that I think it will prove the most valuable topical remedy yet found.

It is this thorough removal of the secretions which is first, last and all the time the most important factor in its successful treatment. After the parts have been thoroughly cleaned, a stimulating application is called for to incite the mucous membrane and glands to a more normal activity. This I usually accomplish with a solution of iodine crystals in glycerine (8 grains to the ounce) applied thoroughly with a soft cotton probe, a method I have found the most efficacious. If the odor is very offensive, it is best to premise the above with a thorough spraying of the cavities with a 2 per cent. solution of camphor-menthol in albolene. As a final step the parts are thoroughly coated with a spray of melted vaseline, which soon congeals and leaves a soft coating upon the atrophic surface. In conjunction with the above, certain instructions must be given the patient to be carried out in the interim at home. I am far from being an advocate of the nasal douche, but in severe forms of this disease I have frequently found it a valuable adjuvant to be used by the patient at home in cleansing the cavities of a large amount of secretion. The danger in its use is at the minimum, since there is no obstruction to the free flow of fluid through the nasal cavities. Let the patient use this douche twice daily (4 Seiler's tablets to a pint of warm water), and afterwards to use thoroughly the spray of aristol in albolene. This method as outlined I have pursued in a large number of cases, and the results so far have been eminently satisfactory. In the mixed variety the treatment differs somewhat on account of the difference existing in the pathological state. In this form we usually have anterior hypertrophi es of the inferior turbinates, and these latter must always be reduced by the galvano-cautery before the after treatment will prove of any success. In any form of the atrophic state all obstructions should be removed which act as barriers to the free exit of nasal secretions. Only of late have I operated upon two cases and removed a septum enchondroma, which was obstructing the free passage of the secretions, to be followed in a short time by a marked improvement of the symptoms. From the above you will rightly conclude that I hold the local treatment of this disease as paramount in importance, but while I do so, I never overlook any systemic dyscrasia that may exist. In young women especially must any irregularities in the menstrual function be corrected, for we must bear in mind that all parts of the human body are largely regulated by the well being of every other part.

